# Estimation of electrical transformer parameters with reference to saturation behavior using artificial hummingbird optimizer

**DOI:** 10.1038/s41598-022-24122-8

**Published:** 2022-11-15

**Authors:** Mohamed F. Kotb, Attia A. El-Fergany, Eid A. Gouda

**Affiliations:** 1 Department of Electrical Engineering, Faculty of Engineering, Mansoura University, Mansoura, Egypt; 2 Department of Electric Power and Machines, Faculty of Engineering, Zagazig University, Zagazig, 44519 Egypt

**Keywords:** Electrical and electronic engineering, Energy grids and networks, Power distribution, Power stations

## Abstract

This paper offers an efficient tool to define the unknown parameters of electrical transformers. The proposed methodology is developed based on artificial hummingbird optimizer (AHO) to generate the best values of the transformer’s unknown parameters. At initial stage, the parameters’ extraction of the transformer electrical equivalent is adapted as an optimization function along with the associated operating inequality constraints. In which, the sum of absolute errors (SAEs) among many variables from nameplate data of transformers is decided to be minimized. Two test cases of 4 kVA and 15 kVA transformers ratings are demonstrated to indicate the ability of the AHO compared to other recent challenging optimizers. The proposed AHO achieves the lowest SAE’s value than other competing algorithms. At advanced stage of this effort, the capture of percentage of loading to achieve maximum efficiency is ascertained. At later stage, the performance of transformers utilizing the extracted parameters cropped by the AHO to investigate the principal behavior at energization of these transformer units is made. At the end, it can be confirmed that the AHO produces best values of transformer parameters which help much in achieving accurate simulations for steady-state and inrush behaviors.

## Introduction

The power transformers are one of the essential and major equipment in power systems. Transformers can transfer energy form generation plants to distribution areas via transmission lines with high efficiency reaches 99% based on its parameters and the related losses^1^. Several research have been introduced to envisage transformer parameters as to minimize its losses, improve its performance and minimize the operational cost. The unknown transformer parameters are nonlinear because of their frequency dependance which makes the transformer modelling accurateness more complex^2^. Transformer parameters estimation became an immense and mandatory challenge for optimal transformer design to realize compulsory standards and specifications^3,4^. The transformer non- linear performance has been addressed as in^2,5^. The determination of transformer unknown parameters is affected by the state of its operation; steady or transient conditions^5,6^. These parameters can be estimated using different methods: the well-known tests; no-load and short circuit tests^7,8^, physical sizing of transformer^9^, manufacturer’s data^10^, and under various load information^7^. Primarily, the analytical methods have been used for fast evaluation of the transformer physical sizing based on finite element analysis (FEA). Recently, non-conventional exploratory and/or evolutionary calculation algorithms have been applied^11^. The evolutionary algorithms have high capability to solve the optimization problems as it can randomly achieve the objective^7^. The optimization methods have been utilized to extract transformer unknown parameters as well as other electrical devices as electric motors, fuel cells, and storage units in addition to find out the electrical operation parameters as optimal load flow and distribution management systems^12–15^. The accuracy of the optimization algorithms is tested by comparing the extracted parameter values against the actual ones^16–18^. A gray box model has been proposed to estimate transformer parameters and study its terminals behaviors at frequencies between 20 kHz to 1 MHz via particle swarm optimization (PSO). This method depends on evaluating the physical dimensions to define winding inductance, capacitance, and loss parameters^6^. The data driven from load testing has been used to extract both single and three phase power transformer parameters via PSO^12^ and Forensic-Based algorithm^1^ has been applied only for single-phase transformer (SPT). Also, slime mold optimizer has been applied to both single and three phase transformers parameter estimation and compared with other optimizers^19^. The 4 kVA SPT parameters have been extracted using the data driven from load testing via Forensic-based investigation and PSO^1^ and bacterial foraging^20^ algorithms and by means of input data through chaotic optimization^7^. The no load losses have been included in the objective function (OF) using manta rays foraging optimizer (MRFO) and chaotic MRFO^3^. Other optimizers have been proposed to evaluate transformer parameters and conducted practical tests for confirmation as coyote optimizer for three and single transformers^21^, and Jellyfish search optimizer, gravitational search algorithm (GSA) and machine learning approach for SPT with 4 kVA rating in^10,22,23^. Multi-objective evolutionary optimization has been adapted to evaluate the transformer parameters, improved using the FEA and verified by comparing the results with the actual measures and behavior^11^. Online transformer parameters evaluation using practical measurements, different slow frequencies and involving transformer turns ratio have been applied to get fast results and save the need for high frequency instruments^24^. Straightforward black-box algorithm through an optimization method with the help of transfer functions estimated by measured voltage ratios has been introduced to extract distribution transformers parameters at frequency between 1 kHz and 1 MHz and in time domain^25^.

Experimental measures of the nonlinear characteristics of inrush current have been used to calculate transformer parameters with the aid of no-load tests with the help of logic function and load tests via PMU in^26^.

Each one of the metaheuristic optimizers has advantages and some difficulties to solve all the problems. Although, genetic algorithm (GA) can solve the multifaceted problems, but it has some drawbacks as it has early convergence, and its precision depends on many selected terms^27^. PSO overcomes the slow convergence, but it has the obstacle of local optima for large scale problems and comparatively affects its control parameters^28^. Ant colony optimizer succeeded in solving dynamic problems, but it has long time for convergence and complicated investigations^29,30^. Artificial bee colony succeeded in making stability between exploration and exploitation but failed in solving the early convergence problem in last iterations and sometimes inaccuracy^31^. Cuckoo search introduced a better performance than PSO and GA for the sophisticated optimization problems^32^ but it has unsatisfactory convergence and local search capability^33,34^.

The main goal of this work is to accurately identify the optimum SPT unknown parameters via new promising metaheuristic optimizer and to study its performance at steady state and inrush conditions. Artificial hummingbird optimizer (AHO)^35^ has been adapted to represent and investigate the SPT. The AHO simulates hummingbirds’ skills and behaviour in looking for its food. It exceeds other meta-heuristic optimizers in reaching objectives with higher precision using lesser control parameters. It has a unique property in its specific environmentalism experience^35^. Zhaoa and el al. have successfully assessed the AHO by applying three tests: fifty mathematical functions with complex characteristics, the IEEE CEC 2014 benchmark functions and ten engineering design problems which proofed the effectiveness of the algorithm^35^.

In this work, the AHO is applied to 15 kVA^7^ and 4 kVA^1^ SPTs, its performance has been studied and compared with other recognized optimizers and works. For more confirmation, the 4 kVA transformer has been simulated using MATLAB/Simulink, comprehensive study has been conducted at steady state and inrush conditions and compared with the calculated well-known behaviour. Also, Interior search algorithm (ISA) has been utilized to investigate the same two cases and held an extensive comparison between the proposed AHO optimizer and other well-known optimizers. The results prove AHO accurateness and its superiority between other optimizers.

The paper contains seven parts: Part 1 declares the Introduction, In Part 2, the SPT mathematical model is introduced. The transformer optimization problem is expressed and adapted in Part 3. In part 4, the AHO procedures are expressed and summarized. The application and assessment of the proposed algorithm to represent SPT and extract its unknown parameters is tested in Part 5. Also, the performance of the proposed algorithm at steady state and at the inrush condition is examined in Part 6. Finally, the remarkable outcomes and conclusions of this search are highlighted in Part 7.

## Mathematical modeling of single-phase transformer and statement

**Figure 1:**
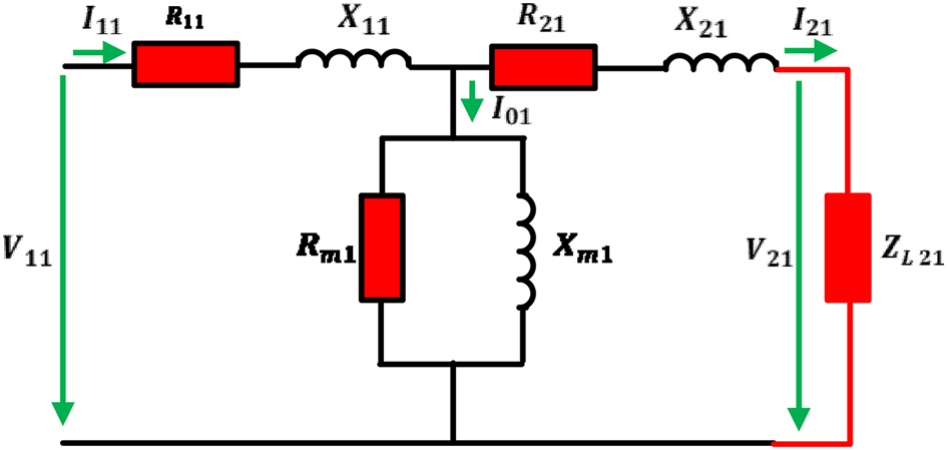
Transformer modelling circuit.

The equivalent circuit of SPT refereed to the primary side is shown in Fig. 1. The modelling contains six variable parameters (${R}_{11}$, ${R}_{21}$, ${X}_{11}$, ${X}_{21}$, ${R}_{m1}$, ${X}_{m1}$) which are described as follows:

The primary and secondary winding’s resistances, primary and secondary reactance’s, core resistance and magnetizing reactance all of them refereed to primary side, respectively^36,37^ as framed in Fig. 1.

The primary windings impedance $\left({Z}_{11}\right)$, secondary windings impedance $\left({Z}_{21}\right)$ and magnetizing impedance $\left({Z}_{m1}\right)$ can be calculated from the following (1) to (3):

1 $${Z}_{11}={R}_{11}+j{X}_{11}$$

2 $${\mathrm{Z}}_{21}={\mathrm{R}}_{21}+{\mathrm{jX}}_{21}$$

3 $${Z}_{m1}=\frac{{R}_{m1}\times j{X}_{m1}}{{R}_{m1}+j{X}_{m1}}$$

If the transformer is loaded by impedance $\left({Z}_{L21}\right)$, with input supply voltage $\left({V}_{11}\right),$ the equivalent transformer impedance referred to the primary side $\left({Z}_{eq1}\right)$, the primary current $\left({I}_{11}\right)$, load current $\left({I}_{21}\right)$, load output voltage $\left({V}_{21}\right)$ and voltage regulation (*ε*) can be formulated by (4) to (8):

4 $${Z}_{eq1}={Z}_{11}+\frac{{Z}_{m1}\times \left({Z}_{L21}+{Z}_{21}\right)}{{Z}_{m1}+{Z}_{L21}+{Z}_{21}}$$

5 $${I}_{11}=\frac{{V}_{11}}{{Z}_{eq1}}$$

6 $${I}_{21}=\frac{{Z}_{m1}}{{Z}_{m1}+{Z}_{L21}+{Z}_{21}}\left(\frac{{V}_{11}}{{Z}_{eq1}}\right)$$

7 $${V}_{21}={Z}_{21}\times {I}_{21}$$

8 $$\varepsilon \%=\frac{\left|{V}_{11}\right|-\left|{V}_{21}\right|}{\left|{V}_{11}\right|}\times 100$$

Also, the input power $\left({P}_{in}\right)$, output power $\left({P}_{out}\right)$, and the corresponding efficiency *η* are given by (9) to (11).

9 $${P}_{in}=real\left\{{V}_{11} .{I}_{11}^{*}\right\}$$

10 $${P}_{out}=real\left\{{V}_{21} .{I}_{21}^{*}\right\}$$

11 $$\eta \% = \frac{{P_{out} }}{{P_{in} }} \times 100$$

The inrush current can be generated due to the change of the transformer magnetization voltage. It can be induced if transformer is energized at no load. The magnitude of inrush current can be considered as high fault current^38,39^. Modelling of the inrush current is mandatory to understand the performance of transformer at energizing operation as has been introduced by Vanti et al.^40^ and expressed as following (12) and (13).

12 $$V\left( t \right) = R_{11} { }i\left( t \right) + L_{11} \frac{di\left( t \right)}{{dt}} + \frac{d\lambda }{{dt}}$$

13 $$i\left(t\right)=\frac{1}{{R}_{p}}\frac{d\lambda }{dt}+{m}_{1}\mathrm{sinh}({m}_{2}\lambda )$$

where ${R}_{11}$ , ${L}_{11}$, and ${R}_{m1}$ are series resistance, series inductance, and the core losses resistance, respectively. ${m}_{1}$ and ${m}_{2}$ are constants which defined the transformer magnetization curve $.$

By considering a sinusoidal source as $V\left(t\right)={V}_{m}\mathrm{sin}(\omega t+\theta )$ the corresponding current can be formulated in time steps as in (14).

14 $$i\left({t}_{j}\right)=i\left({t}_{j-1}\right){e}^{-\rho \Delta \tau }+\frac{{V}_{m}}{{L}_{11}\sqrt{{\rho }^{2}+{\omega }^{2}}}\times \left[\mathrm{sin}\left(\omega {t}_{j}+\theta -\varphi \right)-\mathrm{sin}\left(\omega {t}_{j-1}+\theta -\varphi \right){e}^{-\rho \Delta \tau }\right] +\frac{\gamma }{\rho }\left(1-{e}^{-\rho \Delta \tau }\right)h\left({t}_{j-1}\right)$$

Where $\Delta \tau ={{t}_{j}-t}_{j-1}$ is the time step, $\rho =({R}_{m1}+{R}_{11} )/{L}_{11}$, $\gamma ={R}_{m1}/{L}_{11}$, $\varphi =arctg(\frac{\omega }{\rho })$, and $h\left({t}_{j-1}\right)$ is calculated by (15).

15 $$h\left({t}_{j-1}\right)={m}_{1}\mathrm{sinh}[{m}_{2}\lambda ({t}_{j-1})]$$

The flux calculation is formulated by the formula shown in (16).

16 $$\lambda \left({t}_{j}\right)=\lambda \left({t}_{j-1}\right)-\frac{{V}_{m}}{\omega }\times \left[\mathrm{cos}\left(\omega {t}_{j}+\theta \right)-\mathrm{cos}\left(\omega {t}_{j-1}+\theta \right)\right]+{R}_{11}i\left({t}_{j-1}\right).\sigma+\frac{{R}_{11} {V}_{m}}{{L}_{11}\sqrt{{\rho }^{2}+{\omega }^{2}}}\times \left\{\frac{\mathrm{cos}\left(\omega {t}_{j}+\theta -\varphi \right)}{\omega }-\frac{\mathrm{cos}\left(\omega {t}_{j-1}+\theta -\varphi \right)}{\omega }-\mathrm{sin}\left(\omega {t}_{j-1}+\theta -\varphi \right).\sigma \right\}-\frac{{R}_{11}\gamma h\left({t}_{j-1}\right)}{\rho }\left[\Delta \tau +\sigma \right]-{L}_{11}\left[i\left({t}_{j}\right)-i\left({t}_{j-1}\right)\right]$$

Where

$$\sigma =\left(\frac{{e}^{-\rho \Delta \tau }-1}{\rho }\right)$$

## Optimization of transformer parameters identification

The key goal of the Transformer modeling is to find the proper equivalent circuit to simulate it precisely for imitating practical operations. This should be done at a minimum error level between the experimental and the calculated dataset points. The OF ($${F}_{obj})$$ is defined as minimizing the sum absolute errors (SAEs) among estimated and measured dataset points which is expressed in (17) as follows:

17 $$F_{obj} = minimize\, \left( {\left| {I_{11} - I_{11act} } \right| + \left| {I_{21} - I_{21act} } \right| + \left| {V_{21} - V_{21act} } \right| + \left| {\eta - \eta_{act} } \right|} \right)$$

where ${I}_{11act}$, ${I}_{21act}$, ${V}_{21act}$, and $\eta_{act}$ are the actual data of the primary current, load current, load output voltage, and transformer’s efficiency, respectively. The problem min/max constraints are characterized in (18) for the 6 parameters (${R}_{11}$, ${R}_{21}$, ${X}_{11}$, ${X}_{21}$, ${R}_{m1}$, ${X}_{m1}$) to be optimally extracted.

18 $$\left\{ {\begin{array}{*{20}c} {R_{11 - min} \le R_{11} \left( \Omega \right) \le R_{11 - max} } \\ { R_{21 - min} \le R_{21} \left( \Omega \right) \le R_{21 - max} } \\ {X_{11 - min} \le X_{11} \left( \Omega \right) \le X_{11 - max} } \\ {X_{21 - min} \le X_{21} \left( \Omega \right) \le X_{21 - max} } \\ { R_{m1 - min} \le R_{m1} \left( \Omega \right) \le R_{m1 - max} } \\ {X_{m1 - min} \le X_{m1} \left( \Omega \right) \le X_{m1 - max} } \\ \end{array} } \right\}$$

where,${R}_{11-min}$ and ${R}_{11-max}$ are the min/max values of ${R}_{11}$, ${R}_{21-min}$ and ${R}_{21-max}$ are the min/max of ${R}_{21}$, ${X}_{11-min}$ and ${X}_{11-max}$ are the min/max limits of ${X}_{11}$, ${X}_{21-min}$ and ${X}_{21-max}$ are the min/max of ${X}_{21}$ , ${R}_{m1-min}$ and ${R}_{m1-max}$ are the min/max of ${R}_{m1}$, and ${X}_{m1-min}$ and ${X}_{m1-max}$ are the min/max limits of ${X}_{m1}$,

## AHO optimizer procedure

**Figure 2:**
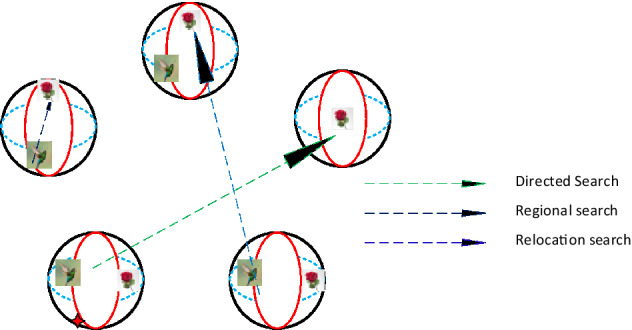
AHO food search classification performance.

AHO is an original optimizer stimulated based on the amazing behaviour of one of the smallest birds all over the world “Hummingbirds”^35^. This bird feeds on mosquitoes, weevils, and aphids. It could shatter its wings with high frequency reaches 80 times/second to follow and hunt its prey. So, the hummingbird is in bad need of plentiful energy that is provided by sucking large amounts of flower syrup and sweetened fluid inside plants. The hummingbird movement, behaviour, skills, and memorization capability have been studied and followed to represent the suggested optimizer. The hummingbird activities can be categorized as three ways for food searching, retention of food resources task and the bird flying forms. A certain number has been proposed for each food resource with a specific plant category. The syrup re-suction rate from the food resource is signified by the value of the OF. The higher fitness value, the higher the syrup re-suction rate and vice versa. Each hummingbird is allocated to a certain location with defined food resource. The location, syrup and sweetened fluid re-suction rate are saved in bird mind and shared with the remaining birds within the population. Also, the location of the unused food resources by the bird can be defined by itself and shared with the others in the population. Each food resource utilization and the last time it was used for a specific bird are registered in a lookup table is initiated and updated. The highest utilization of the food resources is taken as indicator to the better resources to be used for high syrup re-suction rate. AHO search performance can be classified into three major categories; directed food search, regional food search, and relocation food search Fig. 2.

### Initialization

The hummingbird initial location ${LOC}_{i}$ of food supply “i” can randomly be estimated using (19):

19 $$LOC_{i} = L + Ran. \left( {H - L} \right) \quad \forall i \in n$$

where $L$ and $H$ are lower and higher limits for multi-dimensional problem and $Ran$ is the random matrix of values lie between 0 and 1. The food supply lookup table can be initialized as indicated in (20). Null means the hummingbird is fed from its own food supply and zero means the k^th^ hummingbird is fed from j^th^ food supply.

20 $$LUT_{k,j} = \left\{ {\begin{array}{*{20}c} {Null \,if \,k = j } \\ {0\, if\, k \ne j } \\ \end{array} } \right. \forall k \in n\,and\,j \in n$$

### Directed food search

**Figure 3:**
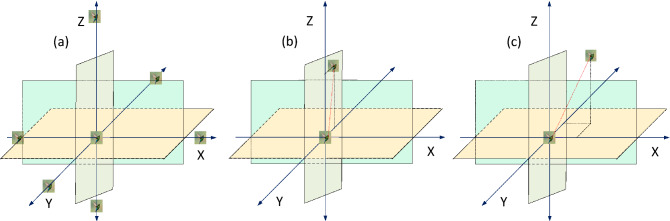
Hummingbirds flying behavior: (**a**) axial fight, (**b**) diagonal flight, and (**c**) omnidirectional flight.

Hummingbird can fly in omnidirectional, diagonal, and axial to look for the food as in Fig. 3. The used directions ${dir}_{i}$ for the food source $i$ can be recorded in a matrix and taken as a guide for the birds as represented in (21).

21 $$dir_{i} = \left\{ {\begin{array}{*{20}c} {Axial \;direction\; flying} \\ {1 \; if \;i = Rand_{i} \left\{ {1,m} \right\}} \\ {else } \\ 0 \\ {\begin{array}{*{20}c} {Diagonal \;direction \;flying} \\ {1 \; if \; i = Q\left( j \right), j \in \left[ {1,p} \right], Q = Rand\_trans\left( p \right), p \in \left[ {2,} \right[Ran1. \left( {m - 2} \right) + 1]} \\ {else } \\ 0 \\ {Single\; direction \;flying} \\ { = 1} \\ \end{array} } \\ \end{array} } \right.$$

where $i \epsilon m$, ${Rand}_{i}\{1,m\}$ and $Rand\_trans(p)$ produce random integer number from 1 to m and from 1 to k respectively but $Ran1$ is a random number from 1 to 0.

Skills of hummingbird leads to reach its food supply goal and nominate the new updated one from the available surrounding sources. The directed food search can be represented as in (22) and (23).

22 $${\upmu }_{i}(IT+1)={LOC}_{i, goal}(IT)+ g.\mathrm{dir}.[ {LOC}_{i}(IT)- {LOC}_{i,goal}(IT)]$$

23 $$g \sim ND(\mathrm{0,1})$$

where ${\mu }_{i}(IT+1)$ is the updated location of the ith food supply at iteration $(IT+1)$, ${LOC}_{i}(IT)$ is the existing location of the i^th^ food supply, and $g$ is the directed factor according to normal distribution $ND(\mathrm{0,1})$ with the mean value zero and the standard deviation one. The general form of the updated location based on the fitness function can be reformulated as in (24).

24 $${LUT}_{i}(IT+1)=\left\{\begin{array}{c}{LUT}_{i}(IT)\, if \,f( {LUT}_{i}(IT)) \le f( {\mu }_{i}(IT+1)) \\ {\mu }_{i}(IT+1)\, if\, f( {LUT}_{i}(IT)) >f( {\mu }_{i}(IT+1))\end{array}\right.$$

Equation (24) shows that the searching for high quality of food resources may start with long distances, but the algorithm tries to go through shorter distances with increasing number of iterations. Hummingbirds is used to transfer to the goal resources with advanced investigations preventing apparent local solutions.

### Regional food search

After sucking the available syrup and food at the existing source, the hummingbird starts to look for other sources in the same surrounding as in (25) and (26).

25 $${\upmu }_{i}\left(IT+1\right)={LOC}_{i, goal}\left(IT\right)+ h.dir.\,{LOC}_{i}(IT)$$

26 $$h \sim ND\,(\mathrm{0,1})$$

where $h$ is the regional factor based on the normal distribution $ND(\mathrm{0,1})$ when the mean value is zero and the standard deviation is one.

### Relocation food search

When the modified location $({\text{MLOC}})$ in the presented region became poor with the food resources, the hummingbird begins to transfer to other locations to search for the required food. Also, if the number of iterations became larger than the relocation factor, the hummingbird located at the positions with lower visit rates will be transferred to a new food resources randomly selected through the hall search area as in (27) and look up table to be revised.

27 $${LOC}_{low-rate}\left(IT+1\right)=L+Ran. \left(H-L\right), \forall\,i\,\epsilon\,n$$

where ${LOC}_{low-rate}$ is the location of the food source with the lower visit rate by hummingbird.

The number of population and iterations are the two important limits affect the process of the AHO. If no replacement food resource is found, the algorithm will start going again to all food resources according to look up table.

At bad conditions, hummingbird may change its food resource goal after two times number of iterations by go to the relocation search stage as per (28).

28 $$RLOCT=2k$$

**Figure 4:**
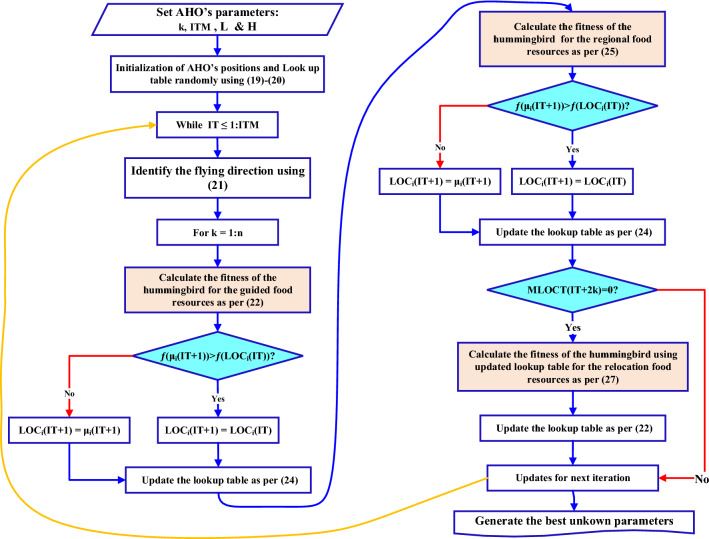
AHO’s flow chart.

The complete procedures of the AHO^35^ are summarized as depicted in Fig. 4.

## Applications and validations

The precision of the AHO optimizer to extract the unknown parameters of SPTs is assessed by applying it to two test cases: 4 kVA and 15 kVA SPTs^1,7^, respectively. For more verifications, the established ISA optimizer is also applied to the two cases and the results of both AHO and ISA compared to other published results in addition to the actual transformers parameters values. Five optimizers are used for the comparisons: PSO^16^, GA^16^, imperialist competitive algorithm (ICA)^10^, chaotic optimization approach (COA) ^10^, and GSA^10^ for test case 1. On the other hand, comprehensive comparisons among PSO^1^, forensic-based investigation (FBI)^1^, JS^21^, GA^17^, ICA^10^, GSA^10^, black-hole optimization (BHO)^41^, and hurricane optimization algorithm (HOA)^42^ to the AHO for test case 2.

To assure the stabilization of the AHO’s performance, 1000 population members, 1000 iterations and 10 independent trials are used using Intel(R) Core (TM) i7-4710HQ CPU@ 2.5GHZ, and 8 GB RAM PC.

### Test case 1

The proposed AHO is executed and applied to 15 kVA SPT based on the nameplate data: 15 kVA, one-phase, 2400 V/240 V, and 50 Hz. The measured transformer currents and voltages at full load are: ${I}_{11act}$ = 6.2 A, ${I}_{21act}$ = 6.2 A, ${V}_{21act}$= 2383.8 V, and *η*_act_ = 99.2%^7,10^.

Comparisons between the transformer parameters obtained by different algorithms previously published results of ICA, GSA, and COA in^10^, and PSO and GA in^16^ are arranged in Table 1. The OF represented by (17) is applied considering the values referred to the primary side of the transformer using standard Z-circuit and short-circuit tests.

**Table 1:** Estimated parameter for the 15 kVA transformer.

Method	${R}_{11}$[Ω]	${X}_{11}$[Ω]	${R}_{21}$[Ω]	${X}_{21}$[Ω]	${R}_{m1}$[Ω]	${X}_{m1}$[Ω]
Actual^5,6^	2.4500	3.1400	2.0000	2.2294	105,000	9106.00
ICA^10^	2.0000	3.0000	1.8000	2.0000	120,000	9200.00
GSA^10^	2.0000	3.1100	1.8100	2.2600	104,281	9094.87
COA^10^	1.9854	2.6117	1.4851	1.5203	131,010	10,074.00
PSO^16^	2.2500	4.0820	2.2000	1.8526	99,517	9009.00
GA^16^	2.7600	3.4140	1.6800	1.8460	97,001	8951.00
ISA	1.5350	5.000	0.81252	2.21821	200,000	10,000.00
AHO	2.2461	5.0000	0.1000	2.2556	200,000	10,000.00

**Figure 5:**
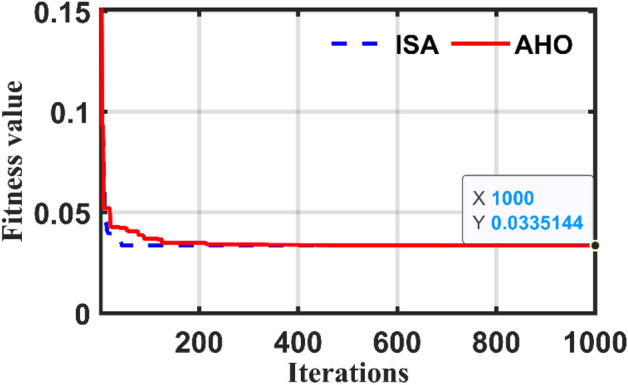
Convergence of the applied optimizers for test case 1.

Table 2 indicates the four terms of the OF resulted from AHO and ISA optimizers against other recognized optimizers. It can be concluded that the errors sum is very close to zero where it is in the range between nearly 6 to 12 for the other optimizers. The results prove the superiority of the proposed AHO-based approach for the parameter estimation of a SPT. For more assessment of the of the applied optimizers, the convergence for case 1 is depicted in Fig. 5. The proposed optimizer produces the minimum SAE’s value which equals to 0.033514. on the other hand, the ISA optimizer error is equal to 0.0335284 which assures the high-quality outcome of the AHO.

**Table 2:** Comparison between the proposed AHO optimizer and other optimizers for 15 kVA transformer.

Variable	ICA^10^	GSA^10^	COA ^10^	PSO ^16^	GA ^16^	ISA	AHO
${I}_{11act}$	6.2000	6.2000	6.2000	6.2000	6.2000	6.2000	6.2000
${I}_{11}$	6.2051	6.2081	6.2079	6.1979	6.1993	6.22572	6.2257
$\left|{I}_{11}-{I}_{11act}\right|$	0.0051	0.0081	0.0079	0.0021	0.0007	0.02572	0.0257
${I}_{21act}$	6.2000	6.2000	6.2000	6.2000	6.2000	6.2000	6.2000
${I}_{21}$	6.1784	6.1781	6.1843	6.1672	6.1678	6.20781	6.20781
$\left|{I}_{21}-{I}_{21act}\right|$	0.0216	0.0219	0.0157	0.0329	0.0322	0.00781	0.00781
${V}_{21act}$	2383.8	2383.8	2383.8	2383.8	2383.8	2383.8	2383.8
${V}_{21}$	2375.5	2375.3	2377.7	2371.1	2371.412.4	2383.8	2383.8
$\left|{V}_{21}-{V}_{21act}\right|$	8.3000	8.5000	6.1000	12.7000	12.4000	0	0
*η*_act_	99.2%	99.2%	99.2%	99.2%	99.2%	99.2%	99.2%
*η*	NR	NR	NR	NR	NR	99.2%	99.2%
$\left| {\eta - \eta _{{act}} } \right|$	NR	NR	NR	NR	NR	0	0
SAEs	8.3267	8.53	6.1236	12.735	12.4205	0.03353	0.03351

*NR*   not reported.

### Test case 2

**Figure 6:**
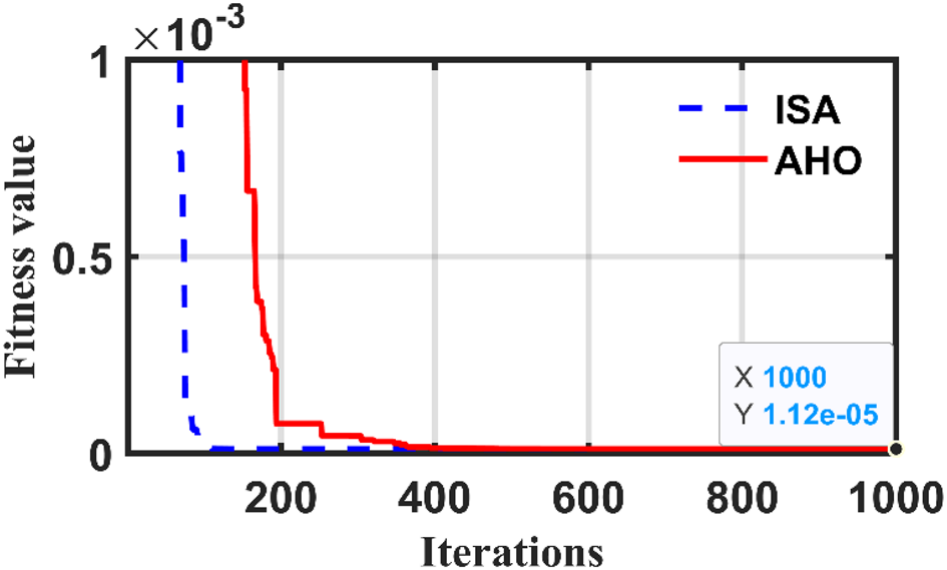
Convergence of the applied optimizers for case 2.

The capability of the proposed AHO optimizer to evaluate the SPT parameters is also tested by applying it to 4 kVA, one-Phase, 250/125 V, 50 Hz and the obtained parameters compared with other documented works as shown in Table 3. Also, the nominal load currents, voltage, and efficiency at full load of the test system are estimated and compared with the well-known optimizers as in Table 4. It can be observed that the value of SAE achieved by the AHO (i.e., 1.12e-5) is the smallest one among the other algorithms. Figure 6 depicts the convergence trends of both AHO and ISA methods for the tested SPT. In addition to that, comprehensive comparisons among PSO^1^, FBI^1^, ICA^10^, GSA^10^, GA^17^, JS^21^, BHO^41^, and HOA^42^ to the AHO as indicated in Tables 3 and 4.

**Table 3:** Comparisons between estimated parameter of AHO and others for 4 kVA transformer at Full load.

Method	${R}_{11}$[Ω]	${X}_{11}$[Ω]	${R}_{21}$[Ω]	${X}_{21}$[Ω]	${R}_{m1}$[Ω]	${X}_{m1}$[Ω]
Actual^1^	0.400	0.200	0.400	2.000	1500.00	750.00
PSO^1^	0.487	0.299	0.326	1.756	1530.00	621.00
FBI^1^	0. 414	0.1722	0.4233	1.725	1508.00	653.00
ICA^10^	0.430	0.202	0.394	2.500	1200.00	700.00
GSA^10^	0.425	0.203	0.415	2.399	1426.00	750.30
GA^17^	0.598	0.226	0.336	1.957	1410.00	707.00
JS^21^	0.405	0.205	0.395	1.987	1520.00	712.00
BHO^41^	0.4512	0.2492	0.378	1.702	1478.78	684.89
HOA^42^	0.4254	0.2017	0.3468	2.1945	1532.90	748.22
ISA	0.3714	0.3727	0.4658	1.4261	1896.27	351.10
AHO	0.3220	0.9030	0.4690	1.234	1407.91	666.32

**Table 4:** Comparisons between errors of the AHO and other optimizers for 4 kVA transformer at Full load.

Variable	PSO^1^	FBI^1^	ICA^10^	GSA^10^	GA^17^	JS^22^	BHO^41^	HOA^42^	ISA	AHO
${I}_{11act}$	15.2825	15.2825	15.2825	15.2825	15.2825	15.2825	15.2825	15.2825	15.2825	15.2825
${I}_{11}$	15.2824	15.282	15.2449	15.2091	15.1714	15.2825	15.2826	15.2825	15.2825	15.2825
$\left|{I}_{11}-{I}_{11act}\right|$	6.54e-4	6.3223e-8	0.0376	0.0734	0.1111	0.0000	1E-04	0.0000	0.0000	0.0000
${I}_{21act}$	15.0782	15.0782	15.0782	15.0782	15.0782	15.0782	15.0782	15.0782	15.0782	15.0782
${I}_{21}$	15.0782	15.0782	14.9881	15.2091	14.9574	15.0782	15.0782	15.0782	15.0782	15.0782
$\left|{I}_{21}-{I}_{21act}\right|$	0.0000	4.5772e-8	0.0901	0.1309	0.1208	0.0000	0.0000	0.0000	0.0000	0.0000
${V}_{21act}$	235.5967	235.5967	235.5967	235.5967	235.5967	235.5967	235.5967	235.5967	235.5967	235.5967
${V}_{21}$	235.5968	235.5967	234.189	234.2083	233.709	235.5967	235.5967	235.5967	235.597	235.597
$\left|{V}_{21}-{V}_{21act}\right|$	4.244e-5	7.1519e-7	1.4077	1.3884	1.8877	0	0	0	0.0003	0.0003
*η*_act_	94.07%	94.07%	NR	NR	NR	94.07%	NR	NR	94.07%	94.07%
*η*	94.01%	94.32%	NR	NR	NR	94.08%	NR	NR	94.07%	94.07%
$\left| {\eta - \eta_{act} } \right|$	0.064	0.255	NR	NR	NR	0.0106	NR	NR	0.0000	0.0000
SAEs	0.0647	0.255	NR	NR	NR	0.0106	NR	NR	1.12e-5	1.12e-5

*NR*  not reported.

## Transformer simulation and performance

**Figure 7:**
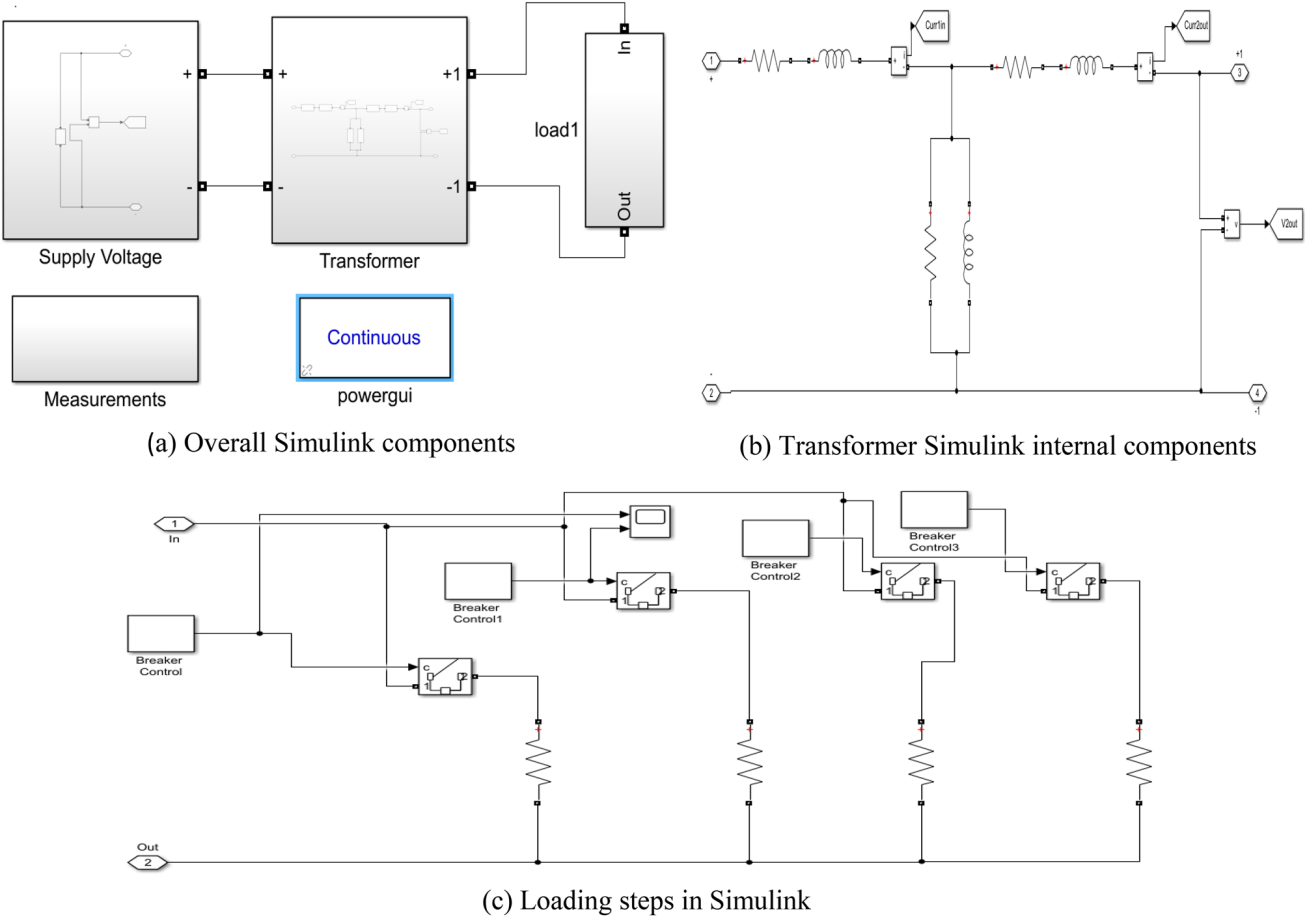
Transformer simulation.

For more proof of the precision of the AHO, the transformer model is simulated in MATLAB/Simulink using the extracted parameters by algorithm as in Fig. 7. The simulation is utilized to study case 2 transformer performance, compare the results with the calculated values, actual performance, and another recognized papers with trusted optimizers at steady state and inrush conditions. Figure 7a–b shows the simulation main components and internal components, respectively while Fig. 7c indicate the transformer simulator resistive loading steps.

### Transformer steady-state operation

**Figure 8:**
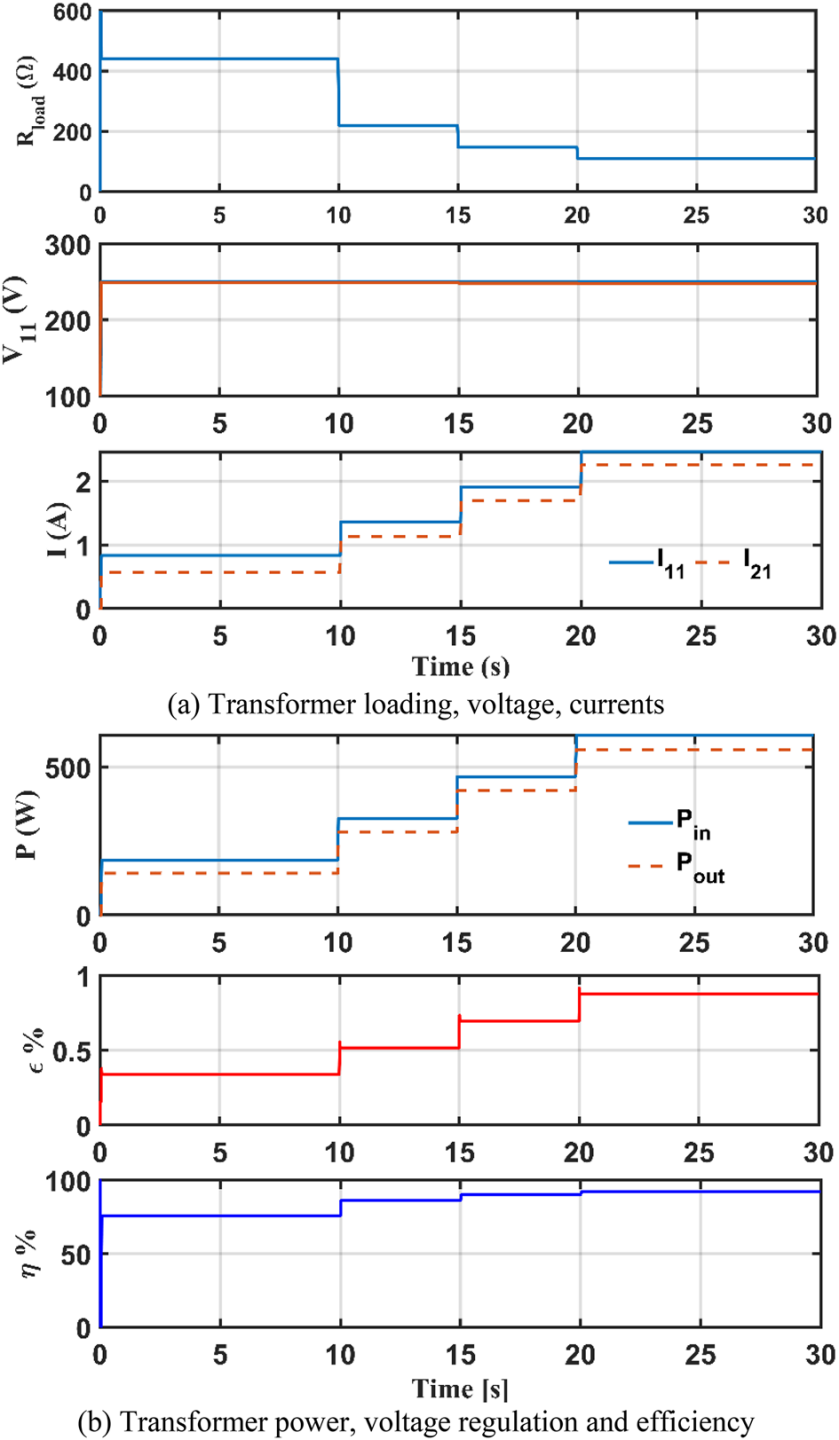
Transformer performance at varied load and UPF as produced by Simulink.

The steady state performance of case 2 transformer with the parameters estimated by the AHO as arranged in Table 3 at unity power factor (UPF) is studied when loading is varied using the mentioned simulator. The effects of load changes on transformer load voltage ${V}_{21}$, current, input/output power, voltage regulation $\varepsilon$ and efficiency *η* are studied as shown in Fig. 8.

**Figure 9:**
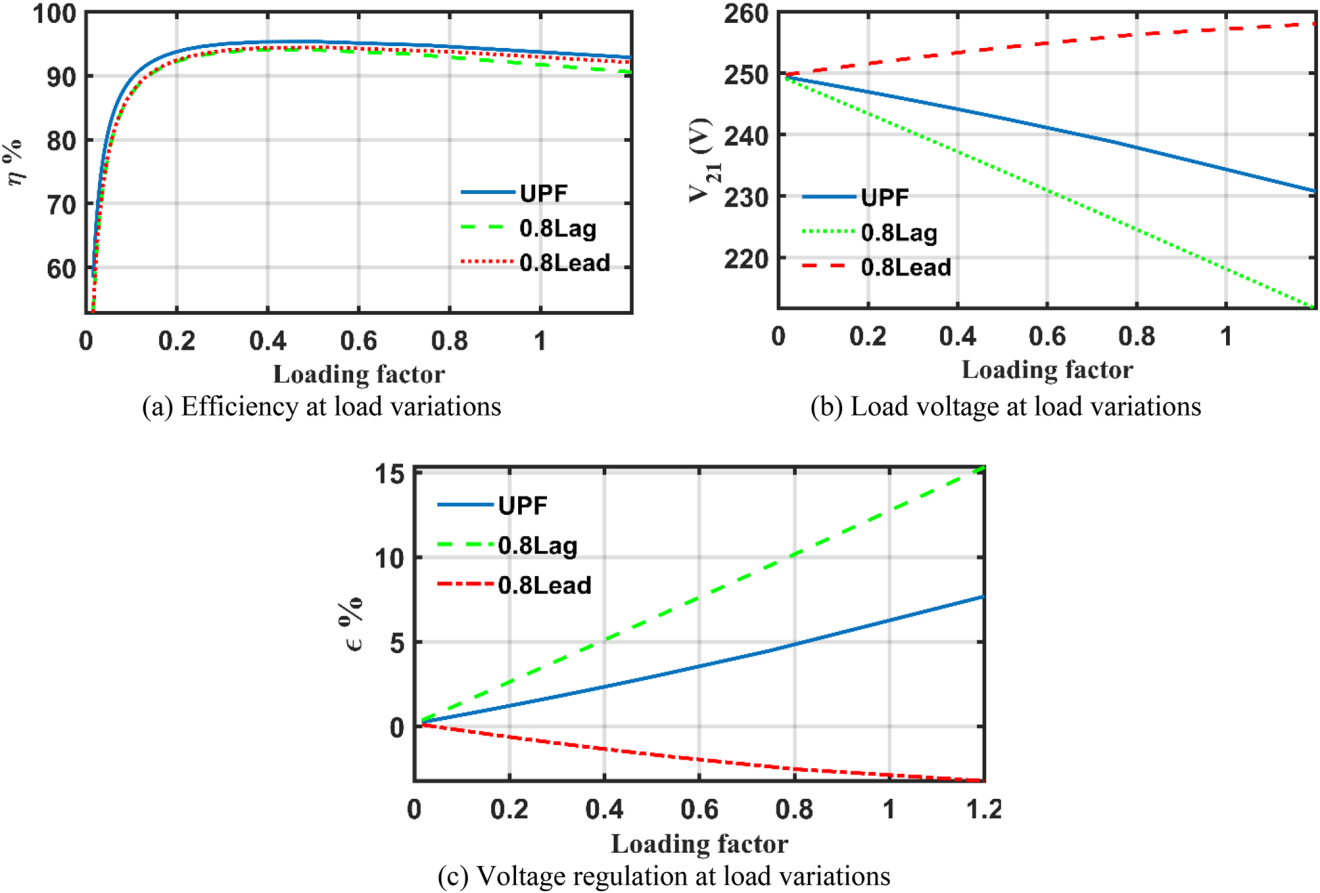
Case 2 transformer performance analysis utilizing AHO’s extracted parameters.

The same data and analysis are done using the mathematical model in "Mathematical modeling of single‑phase transformer and statement" Section considering unity, 0.8 lagging and 0.8 leading power factors and shown in Fig. 9. The transformer performance under unity, and 0.8 lagging PF loading conditions are announced in Tables 5 and 6. The behaviours of *η*, ${V}_{21}$ and *ε* of case 2 transformer using the parameters extracted by the AHO and the simulator is like results calculated by the mathematical model and recognized behaviour which assures the optimizer capabilities. It can be observed from Fig. 9a that the maximum efficiency is achieved at about 45% loading at UPF, and 0.8 lagging PF. As well-known, the transformer loading percentage with respect to full load can be obtained as per (29) and maximum efficiency attained when the cupper losses $\left({P}_{cu}\right)$ is equal to iron losses $\left({P}_{ir}\right)$^43^.

**Table 5:** Case 2 transformer performance for different loading% at UPF.

Loading factor	0.02	0.22	0.306	0.382	0.505	0.746	1.004	1.41
${R}_{Load}$(Ω)	780	70	50	40	30	20	14.6	10
${X}_{Load } ($Ω)	0	0	0	0	0	0	0	0
${V}_{11}$(V)	250	250	250	250	250	250	250	250
${V}_{21}$(V)	249.4	246.7	245.5	244.432	242.61	238.85	234.53	226.91
${I}_{11}$(A)	0.623	3.727	5.108	6.3082	8.285	12.14	16.269	22.9
${I}_{o1}$(A)	0.414	0.413	0.411	0.4102	0.409	0.406	0.4033	0.397
${I}_{21}$(A)	0.320	3.524	4.910	6.1102	8.08	11.94	16.064	22.6
${P}_{cu}$(W)	0.173	10.297	19.713	30.33	52.78	114.39	206.3	410.48
${P}_{fe}$(W)	44.215	43.824	43.641	43.47	43.194	42.59	41.9	40.63
${P}_{in}$(W)	124.1	923.59	1268.78	1567.47	2057.99	3009.58	4015.75	5600.10
${P}_{out}$(W)	79.713	869.47	1205.4	1493.67	1962.0	2852.58	3767.56	5149.0
*η*%	64.232	94.140	95.0	95.29	95.33	94.78	93.8196	91.94
*ε*%	0.257	1.318	1.799	2.227	2.95	4.457	6.1863	9.23

**Table 6:** Case 2 transformer performance for different loading% at 0.8 lagging PF.

Loading factor	0.016	0.174	0.242	0.300	0.396	0.579	0.772	1.078
${R}_{Load}$(Ω)	780.000	70.000	50.000	40.000	30.000	20.000	14.600	10.000
${X}_{Load } ($Ω)	585.000	52.500	37.500	30.000	22.500	15.000	10.950	7.500
${V}_{11}$(V)	250.000	250.000	250.000	250.000	250.000	250.000	250.000	250.000
${V}_{21}$(V)	249.116	244.239	242.145	240.336	237.369	231.614	225.494	215.677
${I}_{11}$(A)	0.651	3.162	4.243	5.174	6.695	9.627	12.715	17.609
${I}_{o1}$(A)	0.414	0.411	0.409	0.408	0.406	0.402	0.398	0.391
${I}_{21}$(A)	0.256	2.791	3.874	4.807	6.330	9.265	12.356	17.254
${P}_{cu}$(W)	0.167	6.875	12.839	19.459	33.230	70.109	123.683	239.516
${P}_{fe}$(W)	44.179	43.460	43.151	42.885	42.450	41.609	40.719	39.304
${P}_{in}$(W)	95.267	595.732	806.506	986.525	1277.690	1828.355	2393.336	3255.878
${P}_{out}$(W)	50.920	545.398	750.517	924.181	1202.010	1716.637	2228.934	2977.059
*η* %	53.450	91.551	93.058	93.680	94.077	93.890	93.131	91.436
*ε*%	0.354	2.304	3.142	3.866	5.052	7.355	9.802	13.729

29 $$Loading\%=\sqrt{\frac{{P}_{ir}}{{P}_{cu}}} * 100$$

A closer look to Tables 5 and 6, it can be observed that the calculated loading ratio at UPF and 0.8 lagging PF nearly equals 45% same as noticed from Fig. 9a which proves the perfection of the results generated by the AHO. To avoid lengthy article, the Table for leading PF is not shown, however, the plot trends are indicated in Fig. 9a–c like other cases with different loading%. The reader may note that the values of voltage regulations have a positive value for UPF and 0.8 lagging and a negative value under capacitive loading (i.e., 0.8 leading) as indicated in Fig. 9c.

**Figure 10:**
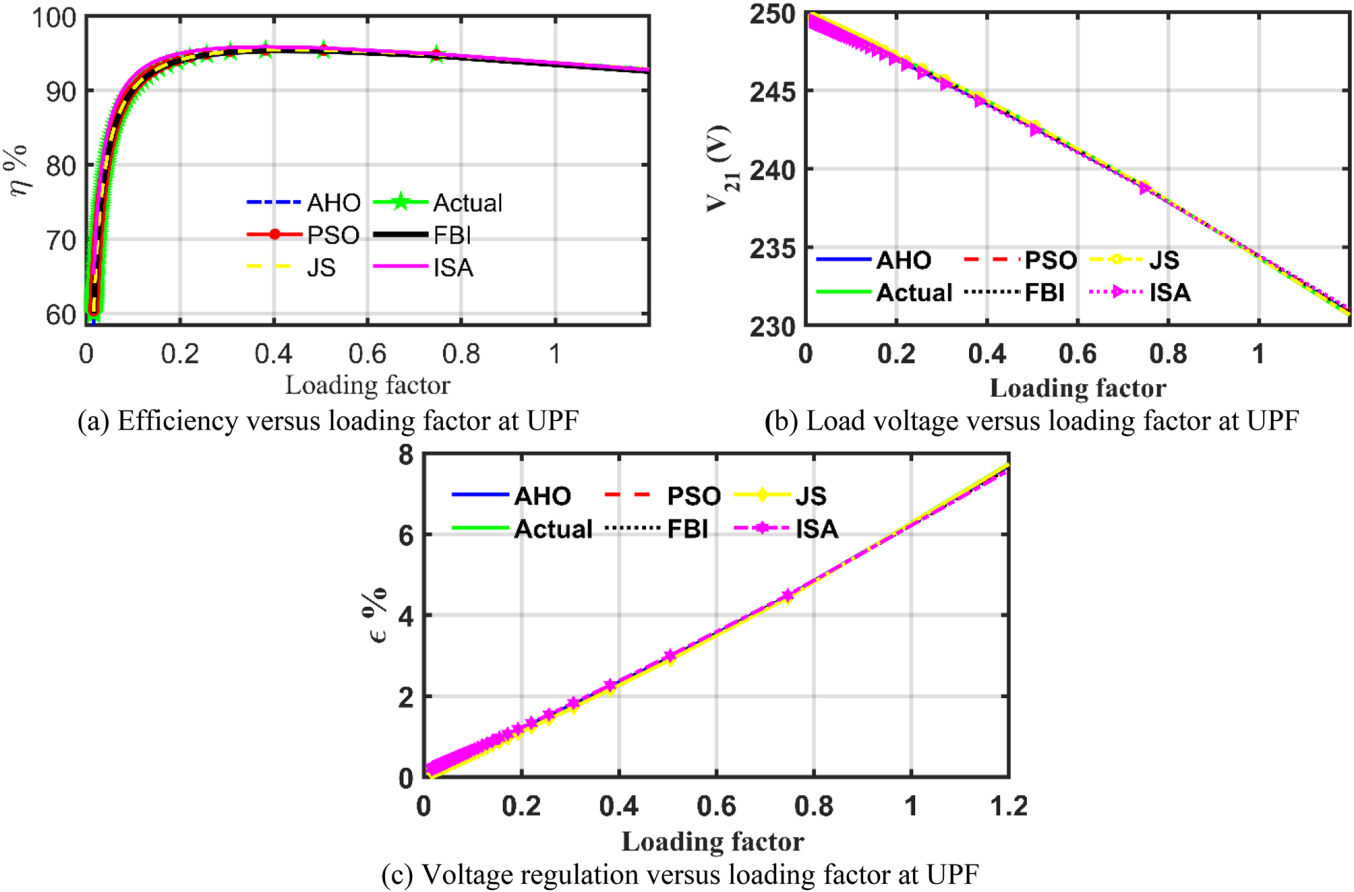
Case 2 transformer performance utilizing the extracted parameters using different optimizers.

The same performance of the mentioned transformer using the parameters estimated by recognized papers using trusted optimizers are compared to the performance using the AHO proposed optimizer as shown in Fig. 10. It can be observed that AHO could not only extract transformer parameters with the lowest error but also lead to performance close to the performance delivered based on the actual readings.

### Transformer operation at inrush conditions

Case 2 transformer inrush current is investigated considering the transformer inrush data in Table 7 including extracted parameters by the AHO. *λ*_0_ is the initial flux linkage value. It is well-known that the study of the inrush current behavior of electric transformer is essential for proper sizing of protective devices to avoid the nuisance tripping during the energization.

**Table 7:** Transformer Inrush terms.

$\Delta \tau$(µs)	${R}_{11}$(Ω)	${L}_{11}$(mH)	${R}_{m1}$(Ω)	${m}_{1}$(mA)	${m}_{2} ({Wb}^{-1})$	$\theta$(°)	*λ*_0_ (Wb)
83.333	0.322364	2.87	1407.91	63.084	2.430	0	0.826

**Figure 11:**
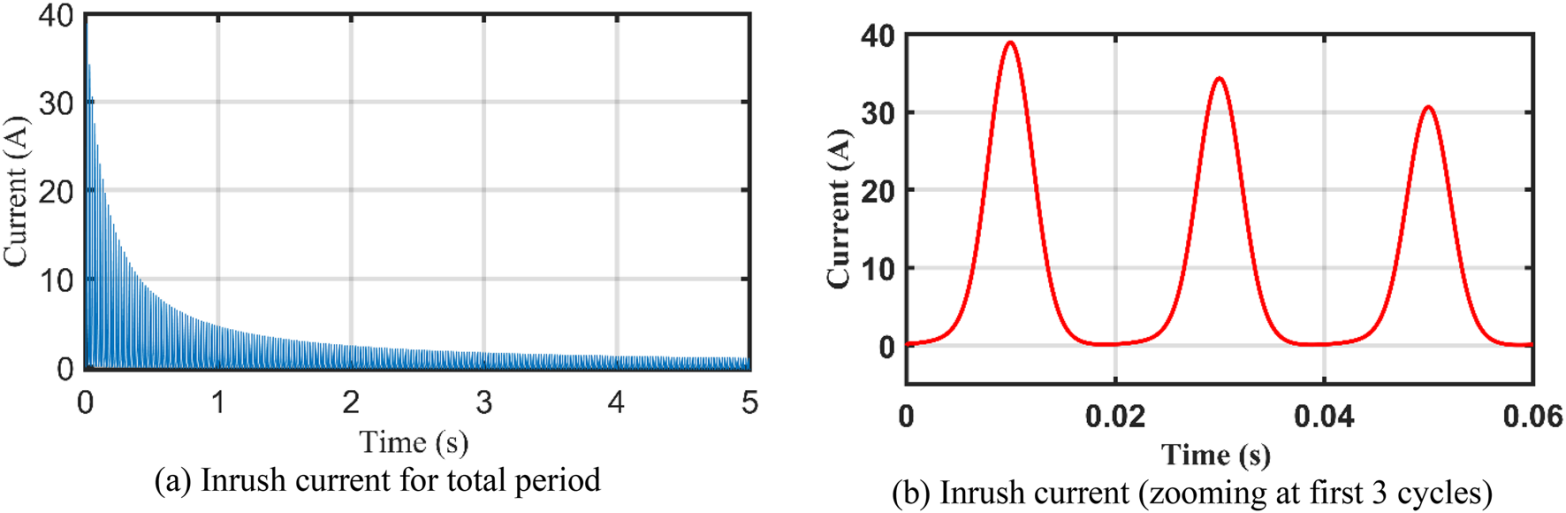
Simulation Inrush current.

**Figure 12:**
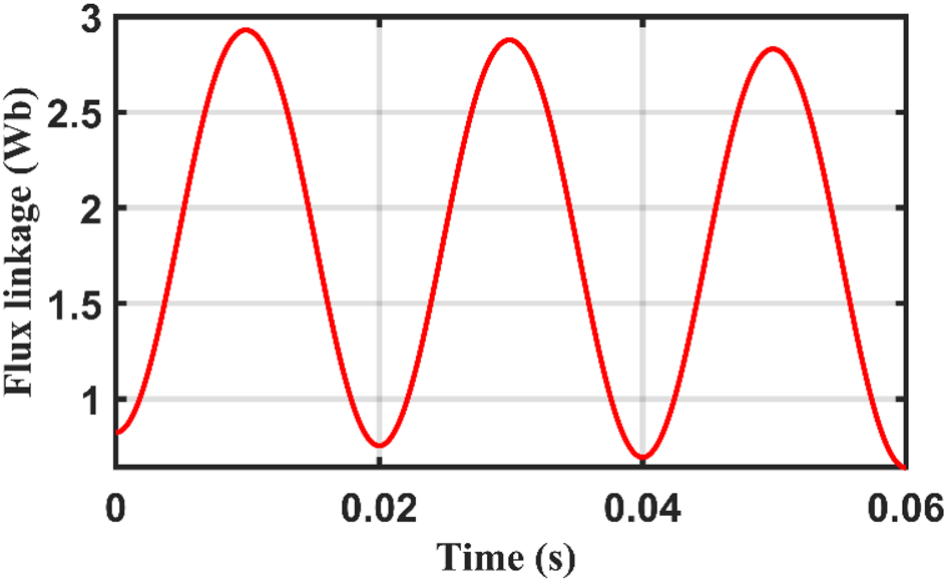
Simulation Flux linkage.

**Figure 13:**
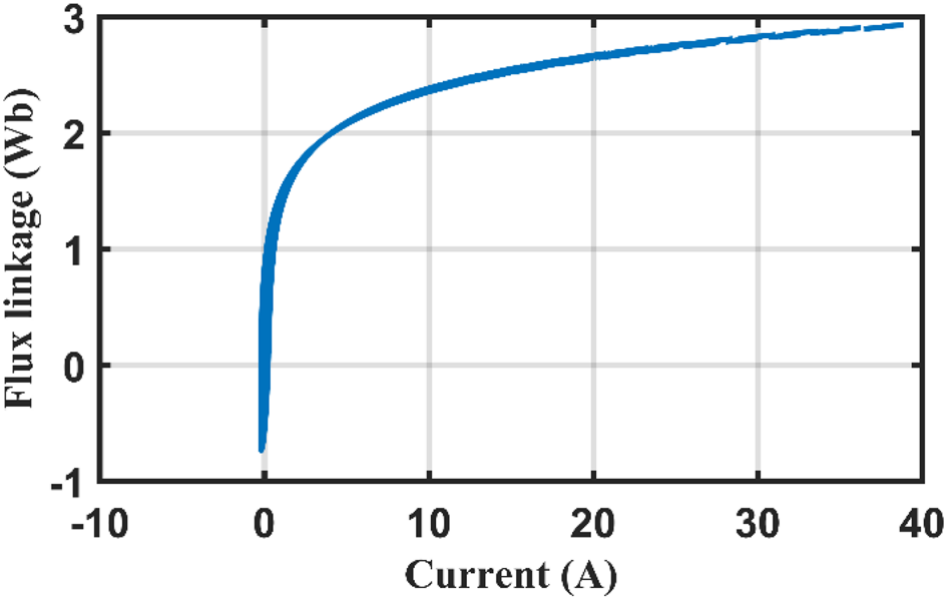
Simulation magnetization curve (flux linkage and current).

**Figure 14:**
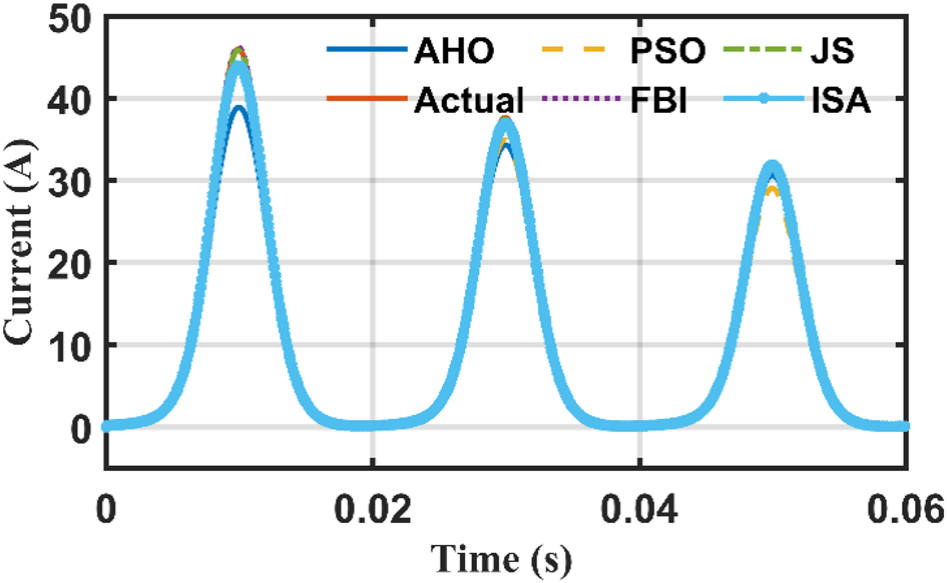
Inrush current behaviour using different optimizers and actual one.

The obtained results for current, flux linkage and magnetization curves are shown at Figs. 11, 12 and 13 and 14 which match the expected inrush performance.

In addition to the above, the inrush current behaviour of the 4 kVA transformer using results of another optimizers (e.g., PSO, FBI, JS, and ISA) are compared against the AHO and actual values as depicted in Fig. 14. It can be observed that all of them are close to the actual inrush behaviour.

### Human and animal rights

This article does not contain any studies with animals performed by any of the authors.

## Conclusions

AHO has been utilized to extract the unknown transformer parameters using its equivalent model. The actual transformer nameplate data is used only to achieve this objective by minimizing the sum of absolute errors (SAEs) among some selected variables. Two test cases have been demonstrated complete with necessary comparisons and further validations. The minimum obtained values of SAEs are 0.033514 and 1.12e-5 for 15 kVA and 4 kVA test cases, respectively. To assure the effectiveness of the proposed tool, the obtained parameters have been used to study the transformer behavior and compared with the well-known performance. The percentage of loading to realize the maximum efficiency has been assigned and the inrush behaviors of transformers have been carried out. In addition to that, the performance of voltage regulations under varied loading conditions at different power factors have been emphasized. The results are very close to the practical ones and proves the efficacy of the proposal. It may be suggested to extend this current work by utilizing the empirical and experimental dataset of large scales of power transformers to define more accurate models including the stray capacitors of the equivalent circuits.

## Data Availability

The data that support the findings of this study are available from the corresponding author upon reasonable request.
